# A pre-therapeutic coating for medical devices that prevents the attachment of *Candida albicans*

**DOI:** 10.1186/s12941-017-0215-z

**Published:** 2017-05-19

**Authors:** Diego Vargas-Blanco, Aung Lynn, Jonah Rosch, Rony Noreldin, Anthony Salerni, Christopher Lambert, Reeta P. Rao

**Affiliations:** Life Science and Bioengineering Center, Worcester Polytechnic Institute, 60 Prescott Street, Worcester, MA 01609 USA

**Keywords:** *C. albicans*, Filastatin, Fungal pathogens, Inhibition of attachment, Biomaterials

## Abstract

**Background:**

Hospital acquired fungal infections are defined as “never events”—medical errors that should never have happened. Systemic *Candida albicans* infections results in 30–50% mortality rates. Typically, adhesion to abiotic medical devices and implants initiates such infections. Efficient adhesion initiates formation of aggressive biofilms that are difficult to treat. Therefore, inhibitors of adhesion are important for drug development and likely to have a broad spectrum efficacy against many fungal pathogens. In this study we further the development of a small molecule, Filastatin, capable of preventing *C. albicans* adhesion. We explored the potential of Filastatin as a pre-therapeutic coating of a diverse range of biomaterials.

**Methods:**

Filastatin was applied on various biomaterials, specifically bioactive glass (cochlear implants, subcutaneous drug delivery devices and prosthetics); silicone (catheters and other implanted devices) and dental resin (dentures and dental implants). Adhesion to biomaterials was evaluated by direct visualization of wild type *C. albicans* or a non-adherent mutant *edt1* ^−/−^ that were stained or fluorescently tagged. Strains grown overnight at 30 °C were harvested, allowed to attach to surfaces for 4 h and washed prior to visualization. The adhesion force of *C. albicans* cells attached to surfaces treated with Filastatin was measured using Atomic Force Microscopy. Effectiveness of Filastatin was also demonstrated under dynamic conditions using a flow cell bioreactor. The effect of Filastatin under microfluidic flow conditions was quantified using electrochemical impedance spectroscopy. Experiments were typically performed in triplicate.

**Results:**

Treatment with Filastatin significantly inhibited the ability of *C. albicans* to adhere to bioactive glass (by 99.06%), silicone (by 77.27%), and dental resin (by 60.43%). Atomic force microcopy indicated that treatment with Filastatin decreased the adhesion force of *C. albicans* from 0.23 to 0.017 nN. Electrochemical Impedance Spectroscopy in a microfluidic device that mimic physiological flow conditions in vivo showed lower impedance for *C. albicans* when treated with Filastatin as compared to untreated control cells, suggesting decreased attachment. The anti-adhesive properties were maintained when Filastatin was included in the preparation of silicone materials.

**Conclusion:**

We demonstrate that Filastatin treated medical devices prevented adhesion of Candida, thereby reducing nosocomial infections.

## Background

Hospital acquired infections are described as “never events”—medical errors that should never have happened. These are largely preventable, serious events that have an adverse effect on public health. The CDC estimates that there are 1.7 million hospital acquired infections each year causing nearly 100,000 deaths, which costs the US healthcare system between 28 billion and 33 billion dollars each year [1]. *Candida albicans*, a normally human commensal organism, has become a significant cause of most nosocomial diseases of fungal origin [2–4]. The number of immunocompromised patients, the population “at risk” and susceptible to fungal diseases [5, 6], steadily increased worldwide at the beginning of the century due to better medical facilities and changes in life style. As expected, this had serious repercussions in the number of reported cases of *C. albicans* infections [7, 8]. In the United States alone, the estimated healthcare cost to treat *C. albicans* systemic infections is between $1.5 and $2 billion per year, which accounts for ∼70% of the total amount spent on systemic fungal infections [9–11]. This is in part due to a reduced number of antifungal drugs, a consequence of the fact that it is difficult to find fungi-specific drug targets that are not also present on host cells. Among the commercially available antifungals, azoles, polyenes and echinocandins are the most effective [12]. In the last few years strains resistant to fluconazole have been reported, and with it a new threat to public health [13–16]. Therefore, new methods to prevent hospital-acquired infections by this opportunistic fungus are becoming more important than ever.

*Candida albicans* is commonly found in the skin and urogenital tract of humans. However, it can become pathogenic causing localized infections such as thrush and vaginitis, the latter being suffered by 75% of females at least once in their lifetime [17, 18]. Furthermore, *C. albicans* can reach the bloodstream and cause systemic infections where the mortality rate can be as high as 50%, even with treatment [19, 20]. Individuals who contract systemic infections caused by this pathogen are typically immunocompromised, such as HIV-infected persons, transplant recipients, patients receiving chemotherapeutic agents, patients receiving large amounts of antibiotics for bacterial infection treatment, and low-birth weight infants [7, 8, 21–24], who are now at an increased risk due to drug resistant *C. albicans* [12, 25–27]. Treating such drug-resistant strains involves long term combination therapy that is often cost prohibitive.

Filastatin was recently identified as a potential agent to prevent *C. albicans* filamentation and adhesion to abiotic and biotic surfaces [10], both of which contribute to biofilm formation and virulence [25, 28–30]. We have previously reported that Filastatin also inhibits the adhesion of *C. dubliniensis*, *C. tropicalis* and *C. parapsilosis* to polystyrene surfaces [10]. Here, we specifically focus on the antiadhesive properties of Filastatin, and propose its use as a pre-therapeutic coating for biomaterials, specifically, dental resin used in dentures and dental implants; silicone elastomers which is widely used as a biomaterial in catheters or as a component of implanted devices that contact the body; bioactive glass which is a component of some medical devices, such as cochlear implants or subcutaneous drug delivery devices that have embedded electronics, and used in prosthetic devices along with titanium to repair and replace diseased or damaged bone [31, 32]. These materials are at high risk of being contaminated with *C. albicans* due to their composition and physical properties [33, 34]. Even more, their common use in clinical settings makes them a suitable reservoir for nosocomial infections [35, 36]. Previous studies have demonstrated, to different extents, the efficiency of coating agents, such as chitosan [37], curcumin on dental resins [38], or the covalent immobilization of the antimicrobials vancomycin and caspofungin on titanium [39] preventing *C. albicans* adhesion and biofilm formation. Thus, we tested various biomaterials under steady-state laboratory conditions as well as physiological flow conditions where the abiotic surfaces were co-incubated or pre-treated with Filastatin. We used analytical techniques such as atomic force microscopy (AFM) to measure the force of adhesion to abiotic surfaces and electrochemical impedance spectroscopy (EIS) to measure the anti-adhesive properties of Filastatin on *C. albicans* under conditions that mimics physiological flow conditions. Finally, we tested silicone material where Filastatin was incorporated into its composition.

## Methods

### Strains and culture conditions

*Candida albicans* isolate, SC5314, obtained from a patient with disseminated candidiasis [40], an mCherry-tagged derivative [41], and the non-adherent mutant *edt1* ^−/−^ [42] were used in this study. *C. albicans* cultures were stored at −80 °C and were propagated on synthetic complete media (SC) agar plates at 30 °C. A single colony obtained from the plates was used to prepare the inoculum in conical tubes containing 10 mL of tryptic soy broth (TSB) that allows cells to grow as planktonic cells. *C. albicans* cells were growth overnight (12–15 h) at 30 °C in a roller drum incubator (64 rpm).

### Preparation of biomaterials

96 well polystyrene plates were used for assay development to test *C. albicans* adhesion. To measure the effect Filastatin wells were pre-treated with Filastatin using 198 µL of diH_2_O + 2 µL of DMSO, or 2 µL of 50 µM Filastatin in DMSO. The wells were washed 10 times in diH_2_O and dried using N_2_ gas. Treated surfaces were visually monitored since the nitrophenyl group on Filastatin absorbs in the visible spectrum (400 ηm).

Glass coupons (1.2 cm diameter) were cleaned using Piranha solution (30 of 35% H_2_O_2_ + 70% H_2_SO_4_) for 1 h, then washed with deionized water for at least 10 min, rinsed with ethanol wash and dried with N_2_ gas. Once dried, the coupons were oxygen plasma cleaned for 2 min, and then silane-coated using 1% APTMS (3-aminopropyltrimethoxysilane) in 96% ethanol (aqueous) for at least 6 h at room temperature and agitated at 60 rpm. The bioactive coupons were finally washed with ethanol, dried with N_2_ gas, and stored at 4 °C until use.

Dental implants resin coupons, made from a fast self-curing acrylic methacrylate resin using the Lang dental Jet Tooth Shade kit, were obtained from Dr. M. Noverr (LSUHSC, New Orleans, LA 70112).

Silicone coupons were made using a mix of polydimethylsiloxane elastomer (PDMS, a silicone elastomer). Briefly, 6 mL of polydimethylsiloxane elastomer were combined with 600 µL of silicone curing agent. After degassing the mix using vacuum for 2 h, the mix was poured on a plane surface and compressed into a fine pellicle of approx. 2 mm of height. Polymerization of the mix took place at 72 °C for 3 h. Coupons of 1.2 cm diameter were then cut from the PDMS pellicle. PDMS coupons including Filastatin were made adding 60 µL of 2.5 mM Filastatin in DMSO to the polydimethylsiloxane elastomer and curing agent mix. PDMS control coupons were made adding 60 µL of DMSO.

Dental resin and PDMS coupons treated with Filastatin prior exposure to cells were placed in a 1.5 mL of diH_2_O containing 50 µM Filastatin in 1% DMSO for 30 min at 37 °C. The coupons were vigorously washed with abundant diH_2_O, dried using N_2_ gas, and stored at 4 °C until use. The presence of Filastatin on the surface of the coupon was confirmed by the evident change of color to yellow.

### Assay development for surface adhesion of *C. albicans* on polystyrene

SC5314 grown overnight were recovered by centrifugation at 1000*g*, 18 °C; washed twice with SC + 0.15%; and adjusted to 0.3 OD_600_ (equivalent to 9·10^6^ cells mL^−1^). Cells were incubated in a polystyrene 96 well plate (198 µL per well) including 2 µL of DMSO (control) or 5, 2.5, or 1.25 mM Filastatin in DMSO for 2.5 h at 37 °C. The wells were emptied and 50 µL of crystal violet 0.5% aqueous solution was added to each well. After 45 min of incubation under static conditions the 96 well plate was washed 10 times with diH_2_O. 200 µL of 75% methanol were added to each well, and the absorbance was read at 590 nm in a plate reader (PerkinElmer, VICTOR^3^) [10]. The absorbance values obtained were used to calculate a relative absorbance. Typically, the relative absorbance was calculated per biological replicate by dividing each sample’s absorbance by the average of the DMSO control absorbance.

For the time-dependent assay, cells were co-incubated with Filastatin following the protocol previously described in 96 well polystyrene plates for varying lengths of time: 5, 15, 30 min, and then every 30 min until 240 min. For Filastatin pre-treated polystyrene, 200 µL of the cell suspension was added to each well. Controls including 1% DMSO or 50 µM Filastatin in 1% DMSO in non-treated wells were also included, as well as a negative control of *edt1* ^−/−^. The 96 well plate was incubated for 4 h at 37 °C, without agitation and light exposure. Adherent cells were also quantified using crystal violet staining following extensive washing. Absorbance was normalized as described above.

### Measurement of surface adhesion of *C. albicans* on various biomaterials

Adhesion was measured using direct cell counts. SC5314 or SC5314 mCherry grown overnight were washed with SC + 0.15% dextrose and adjusted to 0.3 OD_600_. APTMS-treated glass were incubated in a 24 well plates, each well containing 1.5 mL of the cell suspension and 15 µL of 5 mM Filastatin in DMSO, or an equal volume of the solvent as control. Cells were incubated for 2.5 h at 37 °C under static conditions (no agitation). Coupons were then recovered and washed twice with diH_2_O, 10 min each time. Fluorescent images were taken with an Axio Imager Z1 with ApoTome (Zeiss). Twenty images were obtained per coupon, and the cells per imaged field (0.15 mm^2^) were counted using ImageJ v1.49 m [43] and averaged. To visualize the non-tagged SC5314, after washing the coupons the cells were stained with Syto^®^9 and propidium iodide (PI) for 15 min (live/dead assay).

A similar protocol was used for other biomaterials. Briefly, a 24 well plate containing a 1.5 mL of a *C. albicans* cell suspension (0.3 OD_600_) per well was incubated at 37 °C using either APTMS-treated glass coupons, acrylic dental implants coupons, or PDMS coupons in presence and absence of 50 µM Filastatin in 1% DMSO. Acrylic dental coupons and PDMS treated with Filastatin; and PDMS coupons including Filastatin were only incubated with 1.5 mL of *C. albicans* (0.3 OD_600_). The coupons were recovered after 2.5 h, and were gently rinsed with diH_2_O. Then, the coupons were placed in a new plate containing 500 µL of crystal violet per well for a 45 min incubation. Subsequently, the coupons were rinsed in situ using 1000 µL of diH_2_O, three times. The crystal violet stain was recovered from the cells using 500 µL of an aqueous solution of 75% methanol, and the absorbance read at 590 nm.

### Atomic Force Microscopy to measure adhesion force of *C. albicans*

AFM was used to quantify the adhesion force of single *C. albicans* cells to bioactive glass coupons. A 0.3 OD_600_ suspension of SC5314 cells was prepared using SC + 2% dextrose. Two tubes were filled with 2475 µL of cells and 25 µL of DMSO or 5 mM Filastatin in DMSO and incubated at 30 °C in a roller drum incubator. After 30 min the cells were centrifuged at 1000*g* for 5 min and 18 °C, and washed with PBS. A small volume of the cell suspension was pipetted on an agar plate, and using a cantilever coated with Concanavalin A (ConA) a single cell was picked up. This *C. albicans* cell was used for a series of ≥39 adhesion force measurements against a bioactive glass surface, with readings every 5 s using AFM (Asylum MFP-3D-BIO). As an additional control, the non-adherent *edt1* ^−/−^ mutant was also tested for adhesion.

### Electrochemical impedance spectroscopy of *C. albicans* under physiological microfluidic conditions

A microfluidic chamber of 4 mm × 6 mm × 1 mm was fabricated by casting PDMS into an aluminum chamber. Interdigitated micro electrodes (IDE) were fabricated from gold coated glass slides, with the electrode pattern carved using a VersaLaser VLS 2.30 CO2 laser cutter. Each sensor is comprised of a 4 × 6 mm active sensitive surface made of 4 pairs of microelectrodes (400 µm wide with 7.2 mm-long fingers, each finger separated by 400 µm). Before use, the IDE sensors were cleaned with piranha solution, washed for 10 min with abundant diH_2_O, sonicated for 5 min in 96% ethanol, and dried in a stream of N_2_ gas. IDEs were then activated with O_2_ plasma for 2 min and immersed overnight in a 1 mM cysteamine hydrochloride 3% triethylamine ethanoic solution at room temperature. The IDEs were then rinsed with 96% ethanol, submerged in 10% acetic acid in ethanoic solution, and rinsed with 96% ethanol before being dried with N_2_ gas.

A 0.3 OD_600_ suspension of SC5314 in SC + 0.15% dextrose with 50 µM Filastatin in 1% DMSO or 1% DMSO was pumped through the IDEs assembled microfluidic channels (each of 6 mm × 4 mm × 1 mm) at 120 µL min^−1^ for 3 h, and at 37 °C. A syringe pump (KD Scientific) and silicone tubing were used to maintain the continuous flow. EIS measurements were performed every 5 min over a frequency range of 4–100 kHz, 0 V DC bias and a 20 mV p–p sinusoidal excitation signal. Adhesion of *C. albicans* cells over time was measured indirectly by using impedance changes (Zreal at 4 kHz). The experiment was also performed for the non-adherent mutant *edt1* ^−/−^ in presence of the DMSO vehicle (negative control). Each experiment consists of at least three biological replicates. Moreover, a single *C. albicans* cell is regarded as a dielectric component since it consists of biomaterials that have double-layer capacitance [44]. At higher frequencies, the electric current can penetrate the cell body and the impedance is independent of the frequency. At lower frequencies, the double layer capacitance of the cell body and EPS results in a high impedance. The EIS frequency used in biosensing research is usually below 1 MHz, at which there is greater sensitivity to biological bodies, such as cells [45]. In this microfluidic physiological flow [46] experiment, *C. albicans* cells flowing through the IDE were attached and detached from its surface over time due to the nature of the surface, flow shear stress and hydrodynamic forces [47, 48]. As expected, the attachment of cells on the IDE covered the IDE conductive surface and increased the impedance, while the detachment exposed the IDE conductive surface and thus decreased the impedance.

## Results

### Filastatin inhibits *C. albicans* adhesion in a time and concentration dependent manner

**Fig. 1:**
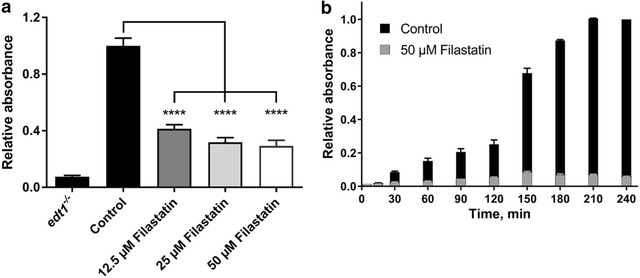
Filastatin-mediated inhibition of *C. albicans* adhesion to abiotic surfaces is **a** dependent on its concentration and **b** increases over time. *C. albicans* were incubated in the presence of Filastatin. Adherent cells were stained with crystal violet and absorbance was measured. Relative absorbance values for each of the three biological replicates and standard error bars displayed, *p* < 0.0001. The *edt1* ^−/−^ mutant lacking an adhesion protein is used as a negative control

We have previously demonstrated that Filastatin inhibits adhesion of many fungal pathogens of the *Candida* spp. to polystyrene surfaces [10]. To further study its anti-adhesion properties we exposed *C. albicans* to increasing concentrations of Filastatin (Fig. 1a). Unbound cells were washed and attached cells were stained with crystal violet to measured absorbance at 590 nm and quantify adhesion. We measured the effect of 12.5, 25 and 50 µM Filastatin compared to the solvent (DMSO) control. Our results indicate that treatment with Filastatin significantly decreases adhesion by 58.7, 68.1 and 70.8%, respectively (p < 0.0001, one-way ANOVA) for *C. albicans*, as compared to the control (Fig. 1a).

In order to determine the exposure time required for Filastatin to trigger an anti-adhesion response in *C. albicans*, we performed a time course experiment from 5 to 240 min using 50 µM Filastatin. Our results showed that the anti-adhesion effect of Filastatin increases overtime (Fig. 1b). Significant reduction of *C. albicans* attachment was observed 15 min post Filastatin treatment as compared to control (DMSO solvent). The effect of Filastatin was exaggerated with increased exposure time: at 15 min post exposure 29.6% of cells were attached, 19.4% after 60 min, and less than a 6% after 240 min. Together these results indicate that effects of Filastatin are concentration and time dependent, and measurable as early as 15 min.

### Filastatin decreases the force of attachment between *C. albicans* and abiotic surfaces

**Fig. 2:**
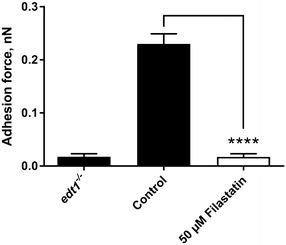
Atomic force microscopy (AFM) to measure adhesion force on *C. albicans* to abiotic surfaces. *C. albicans* cells were lifted with an AFM cantilever and probed against an abiotic surface to measure the adhesion force. The adhesion force of cells treated with Filastatin (*white bar*) is shown in contrast to untreated cells and the *edt1* ^−/−^ non-adherent mutant (*black bars*). Each force measurement is calculated from 35–50 trials, standard error bars displayed, *p* < 0.001. The *graphic* shows one representative experiment

To measure the direct force of attachment of *C. albicans* to an abiotic surface we used AFM. Briefly, cells attached to a surface are probed to measure the force necessary to “pluck” them off the surface [49]. Wild type *C. albicans* was exposed to 50 µM Filastatin and control population was exposed to the solvent (DMSO) for 30 min. The force of attachment for wild type *C. albicans* was recorded at 0.23 nN compared to 0.017 nN when cells were treated with Filastatin (Fig. 2, p < 0.001, one-way ANOVA). This decreased force was comparable to the non-adherent *edt1* ^−/−^ mutant cell attachment to surfaces. These physical measurements further support our previous observations that Filastatin decreases the force of attachment between *C. albicans* and an abiotic surface.

### Filastatin inhibits adhesion of *C. albicans* to a variety of biomaterials

**Fig. 3:**
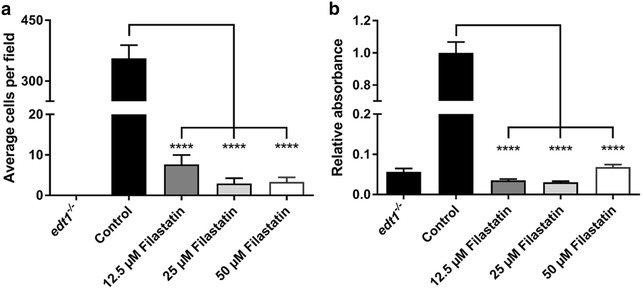
Filastatin inhibits attachment of *C. albicans* to bioactive glass. *C. albicans* was incubated in varying concentrations of Filastatin in 24 well plates containing bioactive glass coupons. **a** Direct cell counts were obtained by propidium iodide and Syto9^®^ staining. *Black bars* represent the number of wild type cells (control) or non-adherent cells (*edt1* ^−/−^) that attach to untreated bioactive glass. *Grey and white bars* represent wild-type cells treated with varying concentrations of Filastatin. Standard error bars displayed, *p* < 0.0001. **b** Crystal violet staining to determine adhered cells to the surface of bioactive glass coupons

To establish the utility of Filastatin in preventing adhesion of *C. albicans* we tested biomaterials such as dental resin, bioactive glass, and silicone. We used 1.2 cm coupons composed of these biomaterials that fit at the bottom of 24 well plates. We standardized the protocol using bioactive glass where we measured adhesion by direct counting of cells attached to the surfaces (Fig. 3a), and spectroscopic measurement of crystal violet stained cells attached to the surface (Fig. 3b). As described earlier we exposed *C. albicans* to varying concentration of Filastatin (12.5, 25 and 50 µM) and allowed cells to attach to bioactive glass coupons. Our results indicate that there was a significant decrease (p < 0.0001) in the number of *C. albicans* cells attached to bioactive glass after treatment with Filastatin as compared to the untreated control (Fig. 3a). Similar results were obtained when adherent cells were stained and the absorbance at A_590_ was measured (Fig. 3b).

**Fig. 4:**
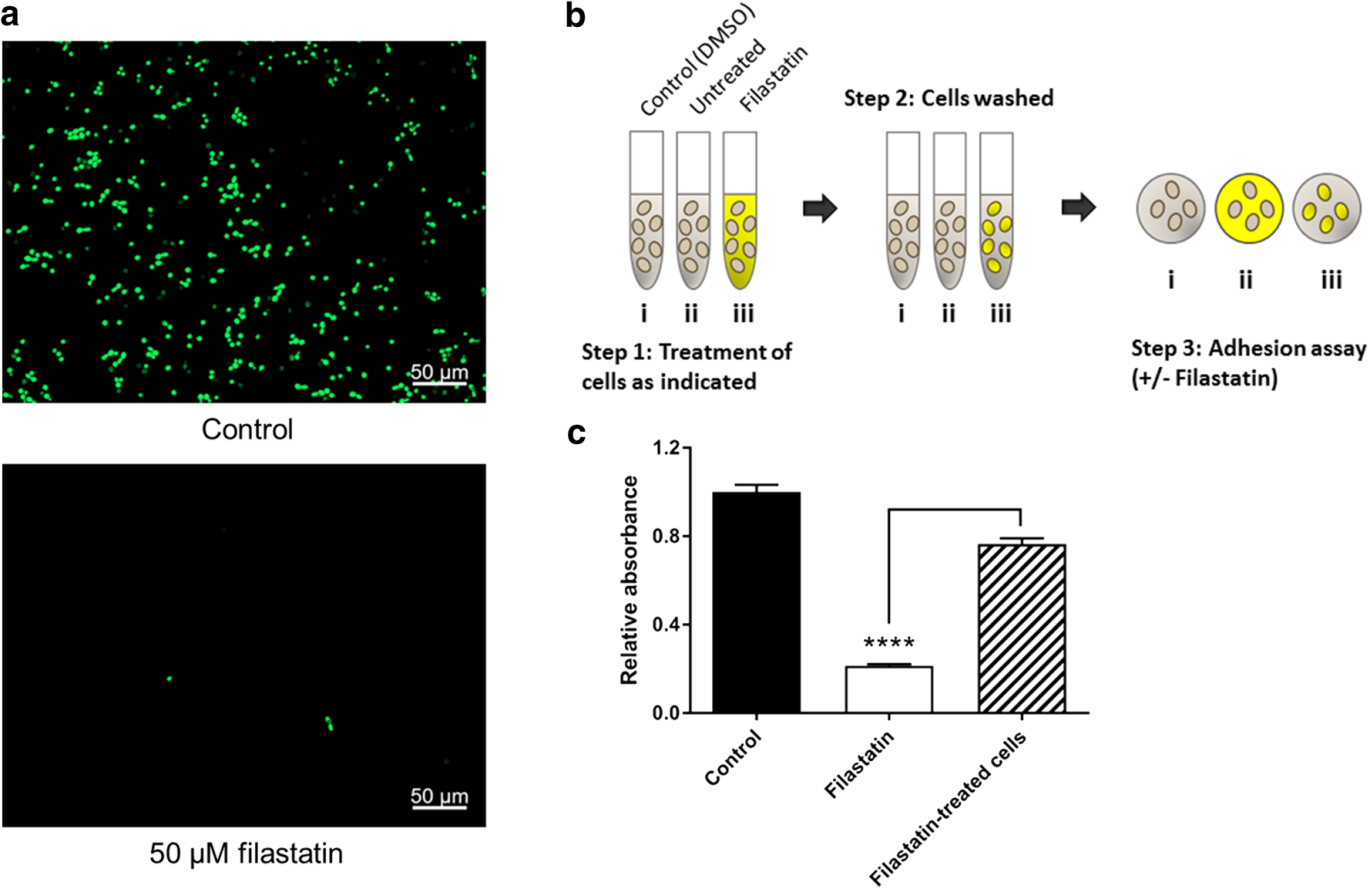
The anti-adhesion effect of Filastatin depends on a continuous exposure to the drug and does not affect *C. albicans* viability. **a** *C. albicans* cells are viable upon exposure to Filastatin. Microscopic imaging of untreated cells attached to bioactive glass (*top panel*, labeled control) indicate that cells were viable but were unable to attach when treated with Filastatin (*bottom panel*). Filastatin treated cells were removed from the incubation media and stained for viability to ascertain that 100% of the cells were accounted for. **b** Experimental design to compare SC5314 cells pretreated with Filastatin vs those under continuous exposure to Filastatin. *Step 1* Cells were incubated with 1% DMSO (*i*), untreated (*ii*), or 50 µM Filastatin in 1% DMSO (*iii*). *Step 2* After 15 min, all the cells were washed three times. *Step 3* Adhesion assay using cell suspensions (*i* control, *ii* continuous exposure to Filastatin, or *iii* pre-treated with Filastatin). *Yellow* represents Filastatin in the media, or cells that were exposed to Filastatin. **c** *C. albicans* cells exposed to Filastatin either as a pre-treatment (*hatched*) or continuously (*white*) were unable to attach to bioactive glass as compared to the untreated cells (*black*). The decrease in adhesion is more dramatic when Filastatin is present continuously. Standard error bars displayed, *p* < 0.0001. Cell adhesion was measured using the crystal violet assay

To determine whether Filastatin affects viability of the cells, we used a live-dead assay (Syto9^®^ and PI) where metabolically active and live cells fluoresce green (Syto9^®^) and dead cells fluoresce red (PI). These studies indicate that adherent *C. albicans* cells treated with Filastatin or those removed from the media containing Filastatin are viable. A representative microscopy image of a coupon surface with fluorescent stained cells is shown in Fig. 4a. To test whether the continuous exposure to Filastatin is needed to disrupt adhesion, we exposed *C. albicans* to 50 µM Filastatin, washed them, and then tested cell adhesion on bioactive glass. The experimental design is outlined in Fig. 4b. Briefly, surface adhesion of *C. albicans* cells was measured after continuous exposure to Filastatin or DMSO compared to that of cells that were washed and recovered in fresh media after Filastatin treatment. Our results (Fig. 4c) indicated that continuous exposure to Filastin decreased surface adhesion by 78% as compared to short exposure to Filastatin where adhesion was decreased by 24%. These results suggest that Filastatin is more effective when present continuously. This prompted us to consider Filastatin as a pre-therapeutic coating for biomaterials.

### Filastatin, can serve as a pre-therapeutic coating for various biomaterials

**Fig. 5:**
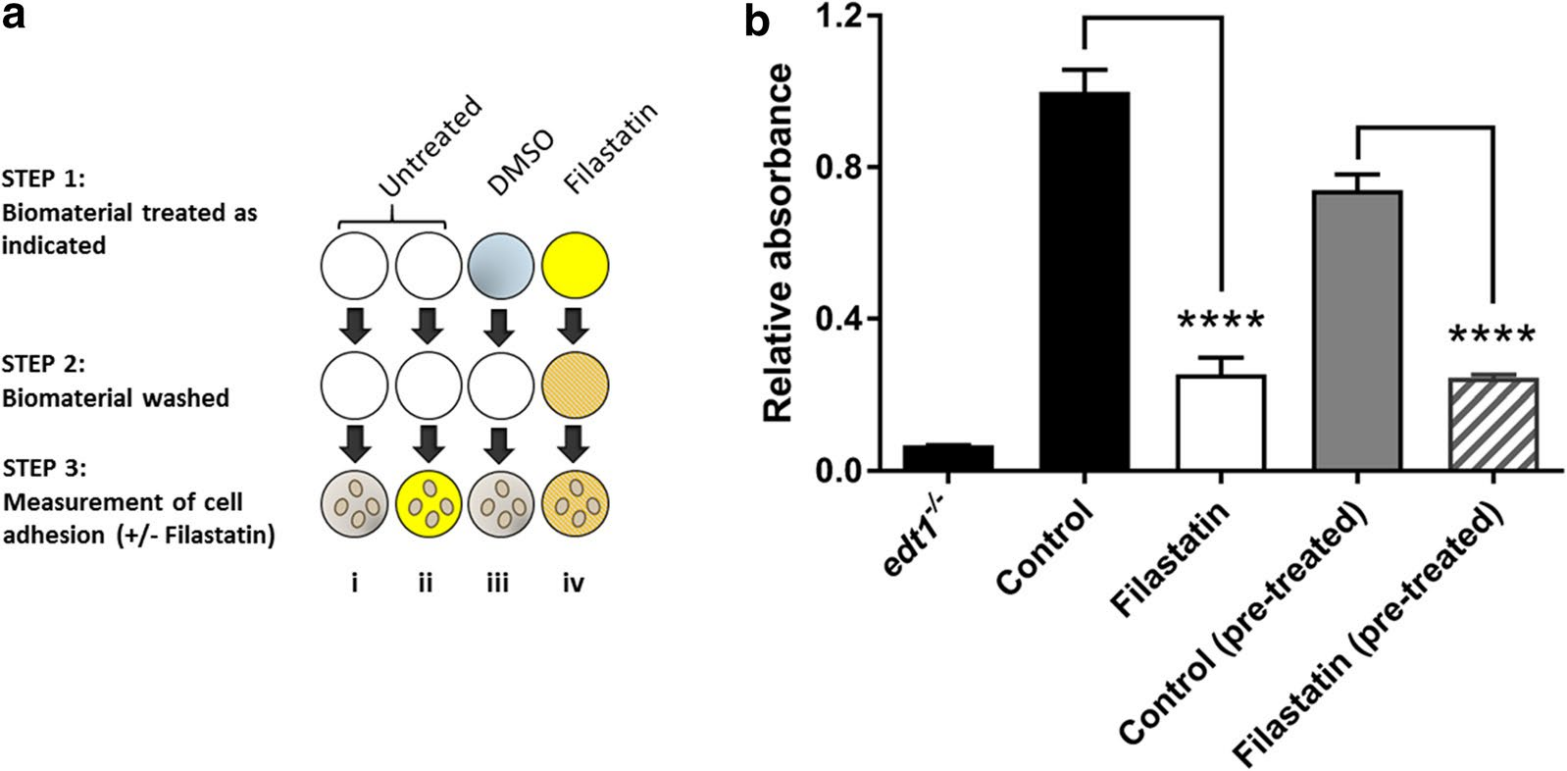
Filastatin-treatment of polystyrene prevents *C. albicans* attachment. **a** Diagram representing the experimental setup. *Step 1* Polystyrene surfaces were untreated (*i*, *ii*), treated with the DMSO solvent (*iii*), or treated with Filastatin (*iv*). *Step 2* Washing. *Step 3* Incubation with *C. albicans* in the presence of solvent (*i*), Filastatin (*ii*), or none (*iii*, *iv*). **b** Crystal violet staining to determine *C. albicans* attachment to Filastatin-treated polystyrene. The *black bars* (control and *edt1* ^−/−^) represent *C. albicans* attachment to untreated polystyrene. The *white bar* represents Filastatin treated *C. albicans* attachment to untreated polystyrene. The *grey bar* represents adhesion of *C. albicans* to polystyrene exposed to the solvent only, and the *grey hatched bar* represents *C. albicans* adhesion to Filastatin-treated polystyrene

Since Filastatin is most effective when present continuously we wanted to test whether it can be used as pre-therapeutic for biomaterials that pose a high risk of contamination with *C. albicans*. *C. albicans* cells incubated on 50 µM Filastatin-treated polystyrene wells showed 24.5% of cell adhesion when compared to the DMSO solvent control. A similar result was obtained when Filastatin was co-incubated (25.37% of cells adhered) with cells. 74% of cells adhered to DMSO solvent treated polystyrene wells, while the positive control *edt1* ^−/−^ mutant strain, registered a 6.77% of cell adhesion (Fig. 5).

**Fig. 6:**
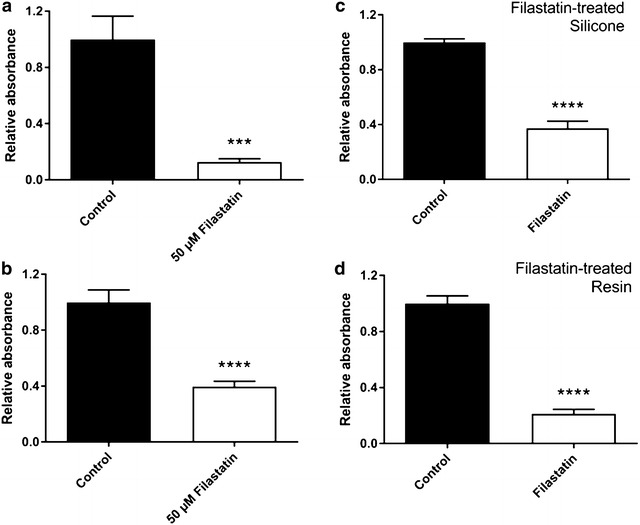
Biomaterials treated with Filastatin prevent *C. albicans* attachment. **a** Adhesion of wild type *C. albicans* cells incubated with or without Filastatin on silicone coupons; or **b** on acrylic dental resin coupons. **c** *C. albicans* incubated on Filastatin-treated silicone coupons, or DMSO-exposed silicone coupons (control); or **d** on Filastatin-treated acrylic dental resin coupons. *C. albicans* adhesion measured using the crystal violet assay. Standard error bars displayed. ***p < 0.001, ****p < 0.0001

Similar decrease in adhesion was observed when silicone surfaces and dental resin were incubated with *C. albicans* cells in presence of 50 µM Filastatin. Cell attachment was decreased by 87.27 and 60.43% as compared to DMSO solvent controls, respectively (Fig. 6a, b).

Silicone and dental resin coupons were also pretreated with 50 µM Filastatin for 30 min prior to exposure to *C. albicans*. Silicone surfaces where Filastatin was chemisorbed showed a 62.7% of reduction of adhesion of *C. albicans*, while similarly treated dental resin showed a 79.7% (Fig. 6c, d). Together these results indicate that biomaterials such as silicone and dental resin when superficially treated with Filastatin prevent attachment of *C. albicans.* These results further establish the utility of Filastatin as a pre-therapeutic for biomaterials. The anti-adhesion effect of Filastatin is lost after treatment with harsh chemicals such as methanol. Binding of *C. albicans* to methanol-treated coupons was similar when compared to untreated resin surfaces. These observations informed our next experiments to incorporate Filastatin into the matrix of biomaterials.

### The anti-adhesive properties of Filastatin persists when incorporated into the chemical composition

**Fig. 7:**
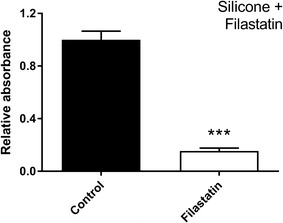
Filastatin incorporated into silicone during formulation confers anti-adhesive properties against *C. albicans*. Crystal violet assay results for *C. albicans* attached to silicone surfaces prepared with Filastatin (*white bar*) or including the DMSO solvent (*black bar*). Standard error bars displayed, *p* < 0.001

To be used as a pre-therapeutic Filastatin may also be incorporated into the composition of the biomaterial. For this purpose, we choose to test silicone since it is a versatile biomaterial specifically used in catheters, a surface that often initiates a candida infection. Our results indicate that incorporation of 25 µM filastatin into the composition of silicone coupons decreased adhesion of *C. albicans* by 6.5-fold when compared to untreated silicone coupons (Fig. 7, p < 0.001). These results demonstrate that Filastatin retains its biological activity when it is incorporated into the silicone material, despite of being exposed at 72 °C for 3 h.

### The anti-adhesive properties of Filastatin persist in physiological flow conditions

**Fig. 8:**
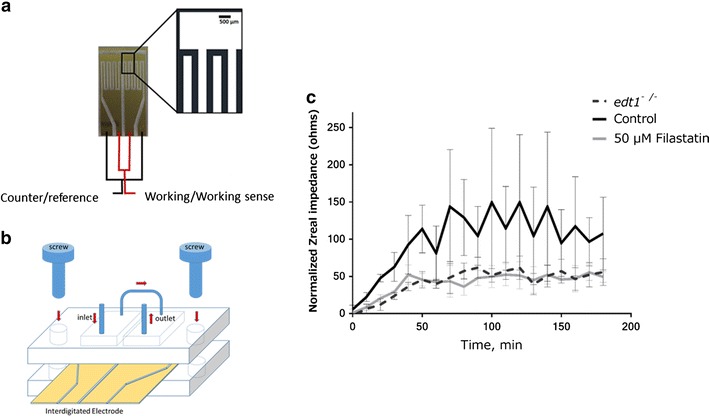
Filastatin prevents the attachment of *C. albicans* under flow conditions. **a** Schematic of the interdigitated gold microelectrodes for sensing impedance upon *C. albicans* attachment. **b** Schematic of the assembly of electron impedance spectroscopy (EIS) device in the microfluidic chamber that mimics physiological flow of bodily fluids. **c** Normalized impedance ratio results for *C. albicans* in SC + 0.15% dextrose and 50 µM Filastatin at 4 kHz. EIS measurements were performed every 10 min using a 20 mV p–p sinusoidal excitation signal. The adhesion over time was measured by using impedance changes (Zreal). SC5314 was used for the positive control and Filastatin treatments, and *edt1* ^−/−^ is the negative control

The effectiveness of Filastatin in a dynamic flow system that mimics physiological conditions was assessed using EIS to measure surface impedance. Higher impedance is indicative of more *C. albicans* cells being attached to surfaces. EIS is widely used in biosensing [50] to measure impedance on amine-terminated IDE (a schematic of the IDE is shown in Fig. 8a, b). Through the course of this microfluidic experiment, the cells experience a continuous flow rate of 120 µL min^−1^ for 3 h, mimicking the conditions in the circulatory system of the mammalian host [46]. *C. albicans* cells continually attached and detached from the surface of the electrode during the experiment. Due to the double-layer capacitance nature of the cells at low frequency range, attachment of the cells on IDE surface is recorded as an increase in impedance changes and detachment of the cells exposes the electrode’s conductive surface and is recorded as a decrease in the impedance [44, 45]. This allows us to measure the impedance as a readout for cell attachment and to determine the effects of Filastatin. *C. albicans* cells treated with Filastatin showed lower impedance as compared to untreated cells. The impedance of the non-adherent mutant *edt1* ^−/−^ was measured as a positive control (Fig. 8c). Indeed, these results indicate that Filastatin is effective under dynamic flow conditions and supports the finding that Filastatin reduces attachment of *C. albicans* to abiotic surfaces.

## Discussion

Over the last few decades the number of cases of systemic candidiasis has increased, outpacing the development of antifungal drugs to fight it. As an opportunistic pathogen, *C. albicans* is responsible for common clinical problems including oral thrush and vaginitis, but can also lead to life-threatening systemic infections in immunocompromised individuals [51], resulting in 30–50% mortality rates [52, 53]. A contributing factor to these statistics is the ability of *C. albicans* to develop resistance to antifungal drugs. In fact, fluconazole-resistant *Candida* strains were more frequently reported [27] and with it the fear of a new threat. The Center for Disease Control (CDC) recently declared drug-resistant *C. albicans* as a major public health threat [13–16, 54]. With the Pharmaceutical industry reporting a low inventory of antimicrobials [55], the search for non-traditional methods to prevent and manage nosocomial fungal infections is becoming more important than ever.

Inhibitors of adhesion are excellent lead compounds for pre-therapeutic and therapeutic drug development because (1) adhesion is the first step of an infection, and (2) inhibition of a general property such as fungal adhesion are likely to have a broad spectrum and affect other fungal pathogens. Efficient adhesion is required for formation of aggressive biofilms, which in turn make *Candida* a successful pathogen [56]. Therefore, adhesion is a pivotal step in fungal pathogenesis, but to our knowledge, one that has not yet been targeted by small molecules.

The discovery of Filastatin and other small molecules with attachment and biofilm disrupting properties [57] can lead to new alternatives for the prevention of nosocomial infections. Here, we further tested the effects of Filastatin on *C. albicans*, showing promising uses as a pre-therapeutic material and even as a composition additive for silicone-based devices.

AFM measurements revealed that Filastatin-exposed *C. albicans* cells were unable to attach to abiotic surfaces and resembled those of the non-adherent *edt1* ^−/−^ mutant cells. The effect of Filastatin is most dramatic when it is continuously present, because cells that were washed to remove Filastatin were capable of binding abiotic surfaces again, albeit with reduced efficiency. These results led us to explore surface-treatment with Filastatin. For this purpose, three biomaterials were tested for co-incubations with Filastatin, and surface-treatment experiments: polystyrene, silicone and acrylic dental resin. In each case, materials treated with Filastatin showed a dramatic decrease in the number of cells adhered and correlated well with results of experiments where cells were co-incubated with Filastatin. The extent of adhesion of cells co-incubated with Filastatin was greater in polystyrene and silicone compared to acrylic dental resin. These results might be caused by DMSO, due to its moderate compatibility/solubility with PDMS and polystyrene [58]. In this present report, the nature of Filastatin attachment to surfaces is ill understood. We conducted preliminary studies using Quartz Crystal Microbalance with Dissipation monitoring technology (QCM-D), which showed Filastatin covering the surface of an APTMS-treated crystal. Future studies will focus on better methods to tether Filastatin to biomaterials.

Our results involving bioactive glass surfaces and plastics confirmed the efficacy of Filastatin as a potent anti-adhesion molecule for *C. albicans* in concentrations as low as 12.5 µM while in co-incubation. Moreover, this effect also extents to *C. dubliniensis*, *C. parapsilosis and C. tropicalis* [10]. Due to its potential use as a pre-therapeutic drug, IES and a microfluidics bioreactor were used to determine the effect of Filastatin under a microenvironment that mimics physiological flow conditions of bodily fluids. The impedance readings can be interpreted as a disruption on the adhesive properties of *C. albicans* cells in the presence of Filastatin, reinstating not only the efficacy of this molecule under steady conditions but also under flow conditions.

It is known that *C. albicans* cell surface interacts directly with host cells and is highly dynamic [59]. Its protein composition changes in the presence of environmental stresses, during switching between budded and hyphal morphologies and also when treated with antifungal agents [59, 60]. Notably, cell surface proteins, nutrient sensing and uptake, morphological switching, and biofilm formation are interrelated factors that contribute directly to the virulence and fitness of *C. albicans* within the host environment [59, 61–63]. Therefore, discovery of compounds that block adhesion, perturb morphological switching and reduce biofilm formation or limit uptake of essential nutrients by *C. albicans* would be an important first step towards developing new antifungal therapeutics.

## Conclusion

We have shown that Filastatin is an effective agent against *C. albicans* adhesion to multiple surfaces under steady and dynamic conditions. Furthermore, *C. albicans* was unable to attach to biomaterials treated with Filastatin. Future studies will focus on the molecular targets of Filastatin and tethering to biomaterials, as well as in safety and of Filastatin as a pre-therapeutic coating.

## Abbreviations

AFM: atomic force microscopy/microscope
APTMS: (3-aminopropyl) trimethoxysilane
CDC: Center for Disease Control
diH_2_O: deionized water
DMSO: dimethyl sulfoxide
EIS: electrochemical impedance spectroscopy
HIV: human immunodeficiency virus
IDE: interdigitated electrode
PDMS: polydimethylsiloxane
PI: propidium iodide
QCM-D: quartz crystal microbalance with dissipation monitoring
SC: synthetic complete (media)
TSB: tryptic soy broth
